# Warming Moxibustion Relieves Chronic Visceral Hyperalgesia in Rats: Relations to Spinal Dynorphin and Orphanin-FQ System

**DOI:** 10.1155/2013/920675

**Published:** 2013-03-16

**Authors:** Li Qi, Hui-Rong Liu, Tao Yi, Lu-Yi Wu, Xi-Ru Liu, Chen Zhao, Yin Shi, Xiao-Peng Ma, Huan-Gan Wu

**Affiliations:** ^1^Key Laboratory of Acupuncture-Moxibustion and Immunological Effects, Shanghai University of Traditional Chinese Medicine, Shanghai 200030, China; ^2^E-Institute of Shanghai Municipal Education Committee, Shanghai University of Traditional Chinese Medicine, Shanghai 201203, China; ^3^Institute of Integrative Medicine, Huashan Hospital Affiliated to Fudan University, Shanghai 200040, China; ^4^Shuguang Hospital, Shanghai University of Traditional Chinese Medicine, Shanghai 201203, China; ^5^Yueyang Hospital of Integrated Traditional Chinese and Western Medicine, Shanghai University of Traditional Chinese Medicine, Shanghai 200437, China; ^6^Shanghai Institute of Acupuncture-Moxibustion and Meridian, Shanghai 200030, China

## Abstract

As a twin therapy of acupuncture in traditional Chinese medicine, moxibustion has shown its effects in relieving abdominal pain in irritable bowel syndrome (IBS) patients and IBS rat models, but its mechanisms are largely unknown. In this paper, we determined the role of spinal dynorphin and orphanin-FQ system in analgesic effect of warming moxibustion (WM) on chronic visceral hyperalgesia (CVH) in IBS-like rat model. Here, we show that (1) repeated WM at bilateral ST25 and ST37 acupoints markedly attenuated the abdominal withdrawal reflex scores in CVH rats; (2) intrathecal administration of **κ** receptor antagonist prior to WM significantly attenuated the WM analgesia and dynorphinA (1-17) enhanced the WM analgesia. WM significantly reinforced the upregulation of spinal dynorphin mRNA/protein and **κ** receptor mRNA levels in CVH rats; (3) intrathecal administration of orphanin-FQ receptor antagonist prior to WM significantly attenuated the WM analgesia and orphanin-FQ enhanced the WM analgesia. WM reinforced the upregulation of spinal orphanin-FQ mRNA/protein and orphanin-FQ receptor mRNA levels in CVH rats. These results suggest that moxibustion may relieve CVH at least in part by activating spinal dynorphin and orphanin-FQ system.

## 1. Introduction

Irritable bowel syndrome (IBS) is a highly prevalent chronic gastrointestinal dysfunctional disease. Chronic visceral hyperalgesia (CVH) has been identified as an important aspect of IBS pathophysiology, and abdominal pain is a primary factor related to the quality of life in IBS patients [1, 2]. Despite multiple therapeutic approaches, the treatment of visceral pain remains a significant challenge [3].

Moxibustion, an important component of traditional Chinese medicine, is a therapeutic method in which burning moxa produces thermal stimulation to the human body, and it affects the function of the meridians and acupoints to prevent and treat the disease [4]. Moxibustion has been adopted as an analgesic method for thousands of years in China and other AsiaN countries and is still frequently used in the present clinical practice because of its advantages of safety, effectiveness, and no side effects [5–7].

Moxibustions include scarring moxibustion (the direct burning of small moxa on the skin), warming moxibustion (also named suspended moxibustion, the direct burning above the skin), and herb-partition moxibustion (indirect burning interposed by various materials) warming moxibustion (WM) is the most practical and convenient one from the perspective of clinical application.

Our previous studies also revealed the analgesic effects of moxibustion in reducing abdominal pain in IBS patients [8, 9] and IBS rat models [10–15]. We also reported that WM treatment could alleviate CVH in IBS-like rats by suppressing hypothalamic corticotrophin-releasing-hormone level [11], which provides the evidence that the analgesic effect of moxibustion may be associated with the central nervous system (CNS).

It has been proved that the afferent signals of viscera could be converged and affected by the signals of superficial body in CNS. This is the essential mechanism of stimulating acupoints for treating visceral disease [16]. The spinal dorsal horn is the first integration center in CNS, and then what role does the spinal cord play in the analgesic effect of moxibustion? What are the kinds of chemicals that participate in the process of CVH and the analgesic effect of moxibustion in IBS-like rats?

Many experimental models of pathological pain, including inflammatory pain [17, 18], neuropathic pain [19], and bone cancer pain [20], show a significant regional elevation of dynorphin and *κ* receptor in the spinal cord. The early research has reported that dynorphin could act on *κ* receptor to impede nociceptive signals in the spinal cord and acupuncture could exert its analgesic action by activating the spinal dynorphin-*κ* opioid system [21, 22]. A variety of pain assays in rodents have shown the effect of intrathecally administered orphanin-FQ (OFQ) to be antinociceptive [23, 24], and it was also reported that acupuncture could exert an analgesic effect by activating spinal OFQ-OFQ receptor system in inflammatory pain model [25].

Both acupuncture and moxibustion are considered to be the external therapy [26], and based on the mechanism of acupuncture analgesia, we presumed that the moxibustion analgesia in CVH rats might be due to the induction of dynorphin and orphanin-FQ system in the spinal cord. We performed the pilot experiment and found that WM could increase the level of spinal dynorphin in CVH rats [13], which holds our interests to further study the spinal dynorphin and orphanin-FQ system in moxibustion analgesia.

Therefore, using a well characterized rat model of IBS-like CVH, the present study was intended to unfold the involvement of spinal dynorphin and orphanin-FQ system in moxibustion analgesia.

## 2. Materials and Methods

### 2.1. Animals

Experiments were conducted strictly in accordance with the National Institute of Health Guide for the Care and Use of Laboratory Animals and the Guidelines of the International Association for the Study of Pain [27]. All efforts were made to minimize the number of animals used and their suffering. Rat model of CVH was induced by mechanical colorectal distention (CRD) during postnatal development. Neonatal male Sprague-Dawley rats (5-day-old) were supplied by the Experiment Animal Center, Shanghai Medical College, Fudan University (permit no.: SCXK (Hu) 2009-0019). Prior to experimental manipulation, rats were allowed to acclimate for 3 days and were maintained under controlled conditions (20 ± 2°C, 60 ± 5% humidity). Every 6–8 neonatal rats were kept with a maternal rat in a cage till 28 days old. The neonatal rats were fed by rat milk, and the maternal rats were fed with food pellets and water ad libitum. 

### 2.2. Induction of CVH and Behavioral Testing for CVH

Neonatal rats were subjected to daily mechanical CRD at the age of 8–21 days. The procedure has been described previously [23]. Mainly, balloon (constructed from a condom, length: 20 mm; diameter: 3 mm) was inserted rectally into the descending colon. The balloon was distended with 0.5 mL air for 1 minute and then deflated and withdrawn. The distention was repeated twice daily at a 30-minute interval. The rats were reared until they reached adulthood (at least 6 week old), and behavioral responses to visceral pain induced by acute CRD were then examined.

Abdominal withdrawal reflex (AWR) scoring system was adopted for behavioral assessment in the evaluation of visceral pain (Table 1), using a procedure as previously described [28]. Briefly, after anesthesia with ether, a balloon (3 cm in length, made using one finger of a latex glove) was inserted into the descending colon. The rats were then housed in a small Lucite cubicles (20 cm × 8 cm × 8 cm) on a platform and allowed to wake up and adapt for 20 minutes. CRD was produced by rapidly inflating the balloon at different levels of CRD pressure (2.66, 5.32, 7.98, and 10.64 kPa) for 20 seconds respectively. Each score was tested three times and there was a 5-minute interval between the two tests to allow the rats to adapt. AWR scores were observed by two observers who were blinded to the experimental conditions. 

### 2.3. Warming Moxibustion Treatment

The detailed WM procedure has been described previously [13]. In brief, warming moxibustion was given to bilateral *TianShu* (ST25, 10 mm lateral to the navel) and *ShangJuXu* (ST37, 5 mm lateral to the anterior tubercle of the tibia and 20 mm below the knee joint), igniting refined moxa stick (Nanyang Hanyi Moxa Co., Ltd., Nanyang, China) (0.5 cm in diameter made of refined mugwort floss) (2 cm high from the acupoints) for 10 minutes, once daily, 7 consecutive days. Rats were not anesthetized before WM treatment, and they were held in a supine position on one gloved hand. Rats of both normal group and model group were also held on one gloved hand in supine position as controls, but not given the WM treatment.

ST25 and ST37 were the two key acupoints chosen in this study based on our clinical treatment of patients with IBS since the 1980s [29, 30]. In the theory of TCM, ST25 is the *mu *point of the large intestine and ST37 is the *xiahe *point of the large intestine, and they are also used for treatment of abdominal pain, diarrhea, and constipation. The two pairs of acupoints (ST25 and ST37) were simultaneously treated with WM. Each treatment consisted of 20 minutes of moxibustion (10 minutes for each pair of acupoints). 

### 2.4. Intrathecal Administration

To investigate the influence of the spinal dynorphin-*κ* system in CRD-induced CVH, i.t. 3.0 nmoL/10 *μ*L dynorphinA (1-17) (Sigma, USA), i.t. 10.0 nmoL/10 *μ*L *κ* antagonist nor-binaltorphimine (nor-BNI, Sigma, USA) 5-minute prior to dynorphinA (1-17), and i.t. 10.0 nmoL/10 *μ*L nor-BNI were performed to the CVH rats at the age of 43 days, respectively. Each injection was followed by 10 *μ*L normal saline (NS) flush. Control experiments were performed by intrathecally injecting CVH rats with NS alone.

To observe the influence of the spinal dynorphin-*κ* system in moxibustion analgesia, 3.0 nmoL/10 *μ*L dynorphinA (1-17), and 10.0 nmoL/10 *μ*L nor-BNI were respectively administered intrathecally to the CVH rats 15-minute before moxibustion at the age of 43–49 days.

To investigate the influence of the spinal OFQ-OFQ receptor system in CRD-induced CVH, i.t. 5 *μ*g/10 *μ*L OFQ (Sigma, USA), i.t. 5 *μ*g/10 *μ*L OFQ receptor antagonist [Nphe^1^]nociceptin(1-13)NH_2_ (Sigma, USA) 5-minute prior to OFQ, and i.t. 5 *μ*g/10 *μ*L [Nphe^1^]nociceptin(1-13)NH_2_ were performed to the CVH rats at the age of 43 days, respectively. Each injection was followed by 10 *μ*L normal saline (NS) flush. Control experiments were performed by intrathecally injecting CVH rats with NS alone. 

To observe the influence of the spinal OFQ-OFQ receptor system in moxibustion analgesia, 5 *μ*g/10 *μ*L OFQ and 5 *μ*g/10 *μ*L [Nphe^1^]nociceptin(1-13)NH_2_ were, respectively, administered intrathecally to the CVH rats 15-minute before moxibustion at the age of 43–49 days.

The procedures of intrathecal injection were as follows: the rat was put into a custom-made organic-glass box with 0.2 mL ether injected for anesthesia. The lower body of the rat was then pulled out from the box with the head kept in the box for sustaining anesthesia. A microsyringe of 25 *μ*L was inserted through the gap between the L4 and L5 vertebrae and extended to the subarachnoid space of the lumbar enlargement. Retain the needle for 10 seconds after injection. Disinfect the local area after withdrawing the syringe. The rat usually come back to conscious within 3 minutes after staying free from ether, which will not disturb the following assessment of pain behavior. Then CVH was assessed within 30–90 minutes after the intervention.

### 2.5. In Situ Hybridization

In situ hybridization (ISH) was utilized to identify the mRNA levels of preprodynorphin (PPD), *κ* receptor (KOR), OFQ, and OFQ receptor. After the behavioral measurement, the rats in each group were deeply anaesthetized with sodium pentobarbital (100 mg/kg, i.p.) and perfused via the aorta with 200 mL NS followed by 200 mL 4% paraformaldehyde in 0.1 M phosphate buffer (PB, pH 7.4). The spinal cord from the L6-S1 segments was removed and postfixed in the fixative solution. Each sample was embedded in paraffin, serially sliced into 5 *μ*m slices. Slices were passed through a dehydrating ethanol gradient and incubated with probe in hybridization solution at a saturated concentration under glass coverslips at 47°C for 18 h. Coverslips were removed in 4× standard sodium citrate and nonspecifically bound probe was removed by treatment with RNase (Sigma, USA) for 30 minutes. Sections were run through stringency washes of 1× SSC and 0.5× SSC at 37°C, and 0.1× SSC at 42°C. The antisense probes were provided by ShangHai DaWeiKe Biotechnology Co., Ltd. The PPD probes are as follows: 5′-AGAGCCTGTCGCAGATGAGGCCGATGAGGTGGAGCAGAAG-3′. The KOR probes are as follows: 5′-GGTGGGCTTAGTGGGCAATTCCCTGGTCATGTTTGTCATC-3′. The OFQ probes are as follows: 5′-ATTCGACGACTTCGTGGTGTCCGTCTGCTTTCAGGCTCCC-3′. The OFQ receptor probes are as follows: 5′-GCGGCGTCAGTTCAAGGTGGTGACTCGGTCCCAGGAG-3′.

### 2.6. Immunohistochemistry

The expression of dynorphin and of OFQ in the spinal dorsal horn was detected by immunohistochemistry. Tissue sections were incubated in primary polyclonal antidynorphin antibody (diluted 1 : 500; Abcam, USA) for 36 hours at 4°C. Then, the bound primary antibody was localized by a biotinylated secondary antirabbit IgG (Boster, CN) incubated for 1 hour in normal goat serum in PBS and subsequently with the avidin biotin complex (ABC kits) at room temperature for 1 hour. Visualization of the antigen-antibody complex was performed using DAB kits (Boster, CN). The same immunohistochemical procedure was followed to determine OFQ (primary antibody: rabbit polyclonal to OFQ 1 : 500, Abcam, USA; secondary antibody: antirabbit IgG 1 : 400). All sections were then cover-slipped with distyrene-plasticizer-xylene (Sigma, USA).

Three sections randomly selected from each animal were captured on the Olympus microscope equipped with a Canon camera. The area and opacity density (OD) of immunoreaction in the spinal cord were measured, analyzed by Motic Med 6.0 (Motic China Group, China), and averaged for sections of each rat and then averaged for the group. Yellow-brown staining granulation was observed with a purple-blue background.

### 2.7. Enzyme-Linked Immunosorbent Assay

Concentrations of spinal dynorphin and OFQ were detected by enzyme-linked immunosorbent assay (ELISA). The L6-S1 spinal cord was removed and stored at −80°C until sonication. Total protein was dissociated mechanically from tissue using an ultrasonic cell disruptor and then centrifuged at 3,000 ×g for 15 minutes. Supernatant was removed and stored at −20°C until analysis. Dynorphin and OFQ were quantified using ELISA kits (RayBiotech, USA) according to the manufacturer's protocol. Measurement was completed using an ELISA with an absorbency maximum at 450 nm.

### 2.8. Statistical Analysis

All data were analyzed using SPSS 11.0 statistical software (SPSS Inc., USA) and expressed as mean ± SEM. One-way ANOVA-LSD test was used if the variance was homogenous. Dunnett's T3 test was used if the variance was heterogenous. *P* < 0.05 was considered statistically significant. 

## 3. Results

### 3.1. Experiment 1: Analgesic Effects of Moxibustion on CVH in IBS-Like Rat Model

**Figure 1:**
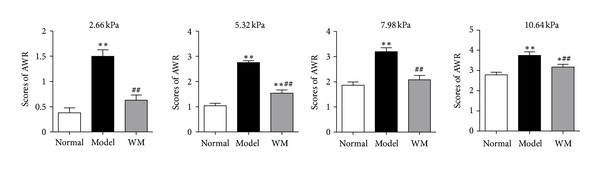
The antiCVH effects of repeated WM in IBS-like rats. WM treatments were performed beginning on the 43rd day and continuing to the 49th day (10 minutes, once daily). Data are presented as the mean ± SEM (*n* = 8 per group). **P < 0.01, *P < 0.05 versus normal group; ^##^ *P* < 0.01 versus model group. WM: warming moxibustion, AWR: abdominal withdrawal reflex.

As shown in Figure 1, at different levels of CRD stimuli (2.66, 5.32, 7.98, and 10.64 KPa), the AWR scores were remarkably higher in model group than that in normal group (*P* < 0.01) and lower in WM group than that in model group (*P* < 0.01), indicating a therapeutic antiCVH effect of WM treatment in IBS-like rats induced by CRD.

### 3.2. Experiment 2: Moxibustion Exert Analgesic Effect by Activating Spinal Dynorphin-*κ* System

**Figure 2:**
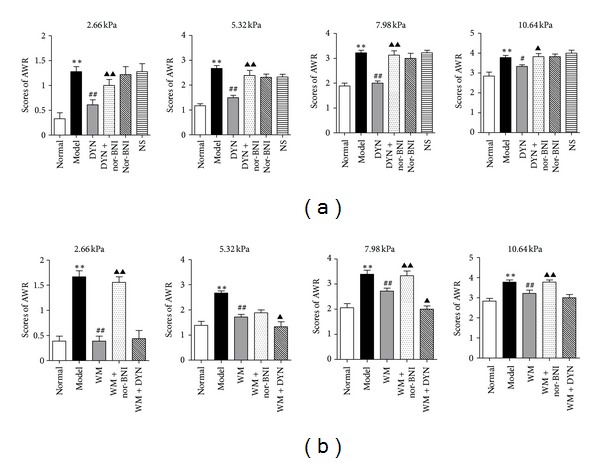
(a) AWR was used for behavioral assessment to evaluation effects of i.t. dynorphinA (1-17) or/and *κ* receptor antagonist on CVH at different levels of CRD stimuli (2.66, 5.32, 7.98, and 10.64 kPa). Data are presented as the mean ± SEM (*n* = 6 per group). **P < 0.01 versus normal group; ^##^ *P* < 0.01, ^#^ *P* < 0.05 versus model group; ^▴▴^ *P* < 0.01, ^▴^ *P* < 0.05 versus DYN group. DYN: dynorphinA (1-17), nor-BNI: *κ* antagonist nor-binaltorphimine. (b) AWR was used for behavioral assessment to evaluation effects of i.t. dynorphinA (1-17) or/and *κ* receptor antagonist on moxibustion analgesia at different levels of CRD stimuli (2.66, 5.32, 7.98, and 10.64 kPa). Data are presented as the mean ± SEM (*n* = 6 per group). **P < 0.01 versus normal group; ^##^ *P* < 0.01 versus model group; ^▴▴^ *P* < 0.01, ^▴^ *P* < 0.05 versus WM group. DYN: dynorphinA (1-17), nor-BNI: *κ* antagonist nor-binaltorphimine, WM: warming moxibustion.

First, we investigated whether the spinal dynorphin-*κ* system could produce inhibitory effect on CVH by a behavioral test. As shown in Figure 2(a), at different levels of CRD stimuli (2.66, 5.32, 7.98, and 10.64 kPa), the AWR scores in model group were obviously higher than in normal group (*P* < 0.01) and in DYN group (*P* < 0.01, *P* < 0.05), and higher in DYN+nor-BNI group than that in DYN group (*P* < 0.01, *P* < 0.05), and there was no significant difference among DYN+nor-BNI, model, and NS group. These data indicated that i.t. dynorphin alleviated the CRD-induced CVH by binding to the *κ* receptor.

Next, we investigated whether the spinal dynorphin-*κ* system was associated with the moxibustion analgesia. As shown in Figure 2(b), at different levels of CRD stimuli, an obvious increase of AWR scores was shown in nor-BNI+WM group compared to WM group (*P* < 0.01), and at stimuli of 5.32 and 7.98 kPa, the AWR scores in DYN+WM group were lower than that in WM group (*P* < 0.05). It suggested that i.t. *κ* antagonist could attenuate the analgesic effect of moxibustion and i.t. dynorphin may enhance it.

**Figure 3:**
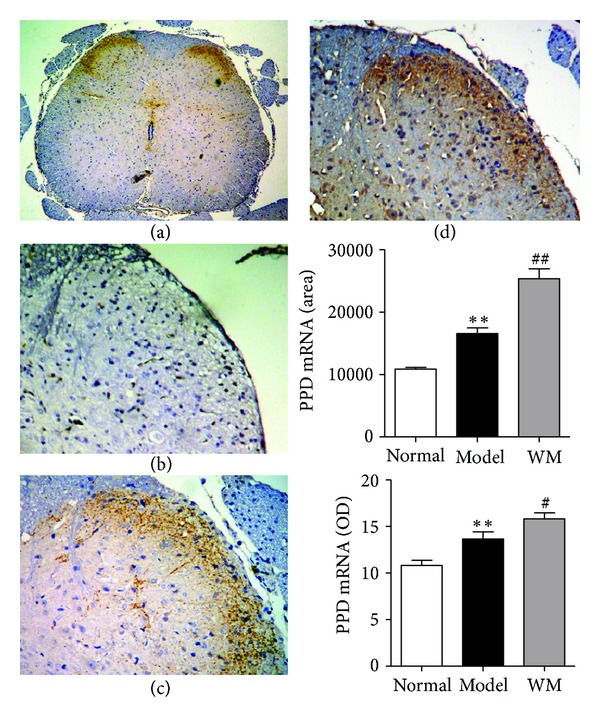
Effects of WM on the expression of PPD mRNA in L6-S1 spinal cord. (a) Spinal dorsal horn from normal group (×40). (b) Spinal dorsal horn from model group (×100). (c) Spinal dorsal horn from model group (×100). (d) Spinal dorsal horn from WM group (×100). Data are presented as the mean ± SEM (*n* = 6 per group). **P < 0.01 versus normal group; ^##^ *P* < 0.01, ^#^ *P* < 0.05 versus model group. WM: warming moxibustion.

**Figure 4:**
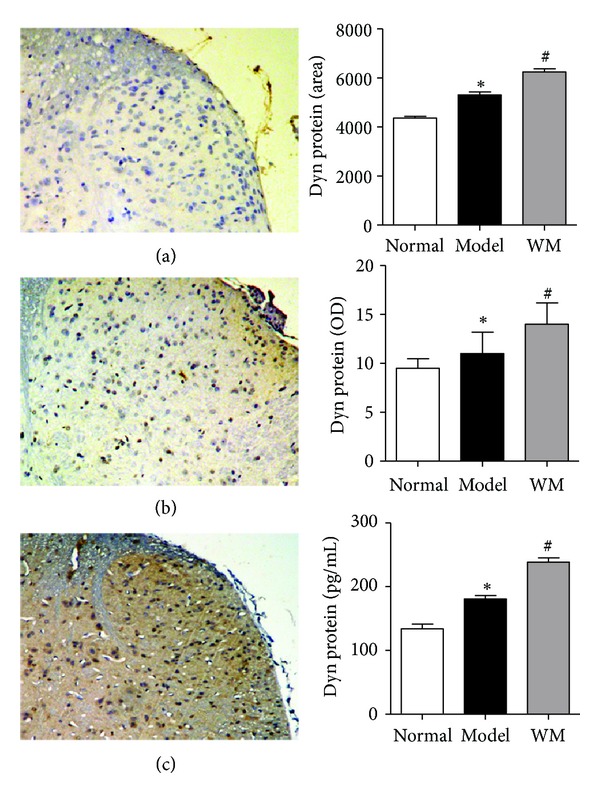
Effects of WM on the expression of dynorphin in L6-S1 spinal cord tested by immunohistochemistry and ELISA. (a) Spinal dorsal horn from normal group (×100). (b) Spinal dorsal horn from model group (×100). (c) Spinal dorsal horn from WM group (×100). Data are presented as the mean ± SEM (*n* = 6 per group). *P < 0.05 versus normal group; ^#^ *P* < 0.05 versus model group. WM: warming moxibustion.

**Figure 5:**
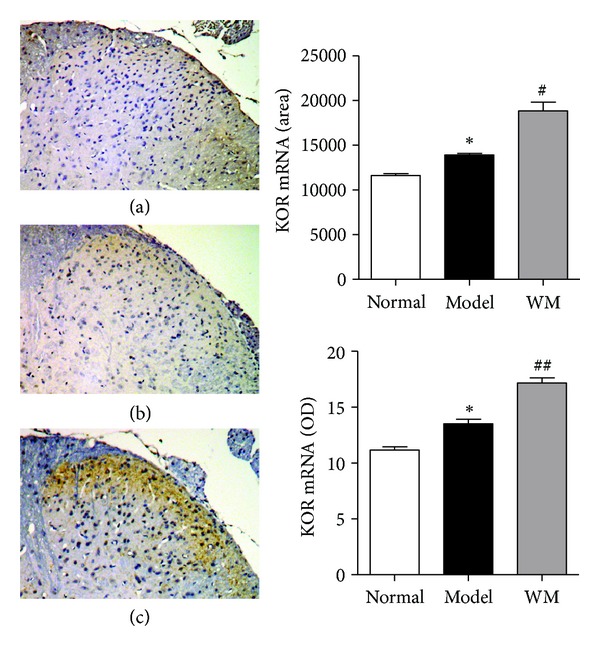
Effects of WM on the expression of KORmRNA in L6-S1 spinal cord. (a) Spinal dorsal horn from normal group (×100). (b) Spinal dorsal horn from model group (×100). (c) Spinal dorsal horn from WM group (×100). Data are presented as the mean ± SEM (*n* = 6 per group). *P < 0.05 versus normal group; ^#^ *P* < 0.05, ^##^ *P* < 0.01 versus model group. WM: warming moxibustion.

We further tested the expression of dynorphin and *κ* receptor in L6-S1 spinal dorsal horn by ISH, immunohistochemistry, and ELISA. The expression of PPD mRNA in model group was higher than that in normal group (*P* < 0.01), and WM treatment further produced a marked elevation of PPD mRNA (*P* < 0.01, *P* < 0.05) (Figure 3). At the protein level, the expression of dynorphin-immunoreaction was higher in model group than that in normal group (*P* < 0.05), and dense immunoreactivity for dynorphin was shown after WM treatment (*P* < 0.05). Similarly, the results of the ELISA showed that the dynorphin increased remarkably during CVH and was further enhanced by WM treatment (*P* < 0.05) (Figure 4). ISH showed that the expression of spinal KOR mRNA increased during CVH and was further remarkably enhanced by WM treatment (*P* < 0.01, *P* < 0.05) (Figure 5). These data indicated that WM treatment induced the expression of dynorphin and enhanced the transcription of KOR mRNA in CVH rats.

### 3.3. Experiment 3: Moxibustion Exert Analgesic Effect by Activating Spinal OFQ-OFQ Receptor System

**Figure 6:**
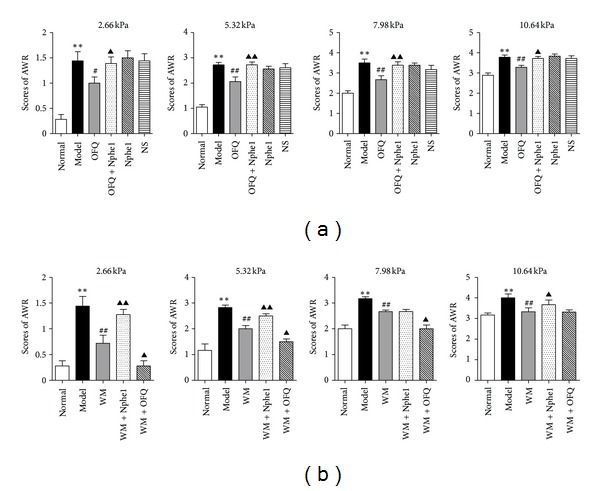
(a) AWR was used for behavioral assessment to evaluation the effects of i.t. orphanin FQ or/and OFQ receptor antagonist [Nphe^1^]nociception(1-13)NH_2_ on CVH at different levels of CRD stimuli (2.66, 5.32, 7.98, and 10.64 kPa). Data are presented as the mean ± SEM (*n* = 6 per group). **P < 0.01 versus normal group; ^##^ *P* < 0.01, ^#^ *P* < 0.05 versus model group; ^▴▴^ *P* < 0.01, ^▴^ *P* < 0.05 versus OFQ group. OFQ: orphanin FQ, Nphe^1^: OFQ receptor antagonist [Nphe^1^]nociceptin (1-13)NH_2_. (b) AWR was used for behavioral assessment to evaluate the effects of i.t. orphanin FQ or/and [Nphe^1^]nociception(1-13)NH_2_ on moxibustion analgesia at different levels of CRD stimuli (2.66, 5.32, 7.98, and 10.64 kPa). Data are presented as the mean ± SEM (*n* = 6 per group). **P < 0.01 versus normal group; ^##^ *P* < 0.01 versus model group; ^▴▴^ *P* < 0.01, ^▴^ *P* < 0.05 versus WM group. OFQ: orphanin FQ, Nphe^1^: OFQ receptor antagonist [Nphe^1^]nociception(1-13)NH_2_, WM: warming moxibustion.

As shown in Figure 6(a), at different levels of CRD stimuli (2.66, 5.32, 7.98, and 10.64 kPa), the AWR scores in model group were higher than that in OFQ group (*P* < 0.01, *P* < 0.05). The AWR scores were higher in OFQ+Nphe^1^ group than that in OFQ group (*P* < 0.01, *P* < 0.05). These data indicated that i.t. OFQ alleviated the CRD-induced CVH by binding to the OFQ receptor.

As shown in Figure 6(b), at different levels of CRD stimuli, an obvious increase of AWR scores was shown in Nphe^1^+WM group compared to WM group (*P* < 0.01, *P* < 0.05), and the AWR scores in OFQ+WM group was lower than that in WM group (*P* < 0.05). It suggested that i.t. [Nphe^1^]NC(1-13)NH_2_ could attenuate the analgesic effect of moxibustion on CVH and i.t. OFQ could enhance it.

**Figure 7:**
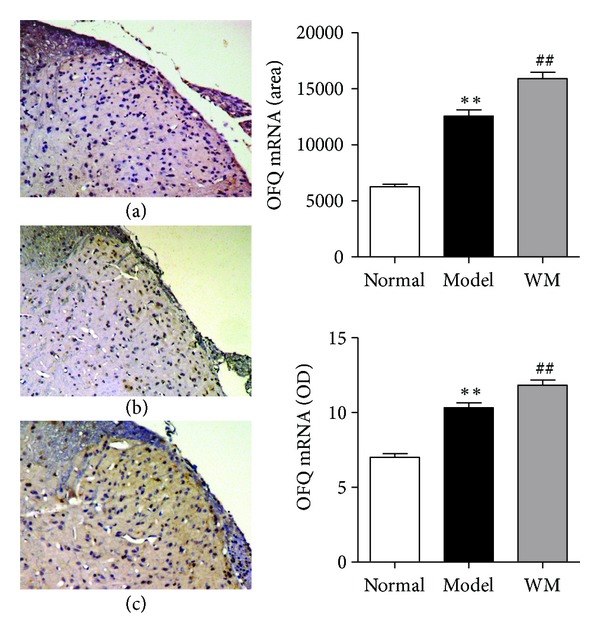
Effects of WM on the expression of OFQ mRNA in L6-S1 spinal cord. (a) Spinal dorsal horn from normal group (×100). (b) Spinal dorsal horn from model group (×100). (c) Spinal dorsal horn from WM group (×100). Data are presented as the mean ± SEM (*n* = 6 per group). **P < 0.01 versus normal group; ^##^ *P* < 0.01 versus model group. WM: warming moxibustion.

**Figure 8:**
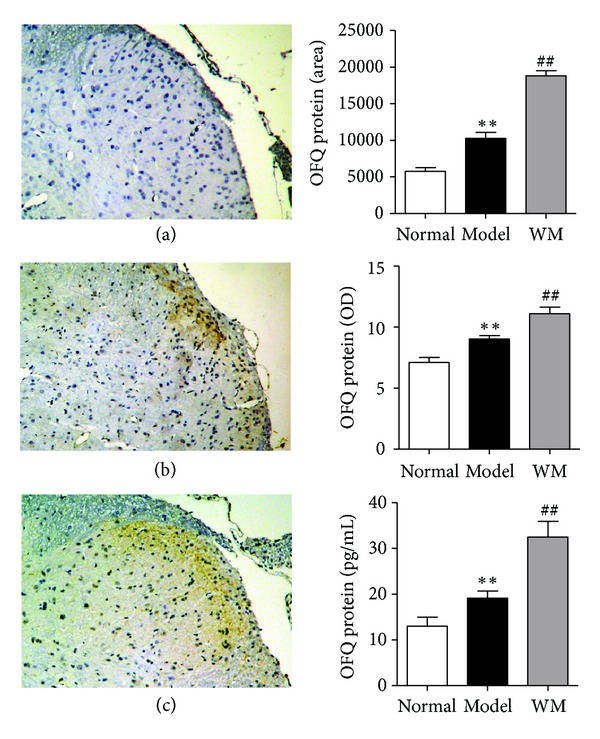
Effects of WM on the expression of orphanin FQ in L6-S1 spinal cord tested by immunohistochemistry and ELISA. (a) Spinal dorsal horn from normal group (×100). (b) Spinal dorsal horn from model group (×100). (c) Spinal dorsal horn from WM group (×100). Data are presented as the mean ± SEM (*n* = 6 per group). **P < 0.01 versus normal group; ^##^ *P* < 0.01 versus model group. WM: warming moxibustion.

**Figure 9:**
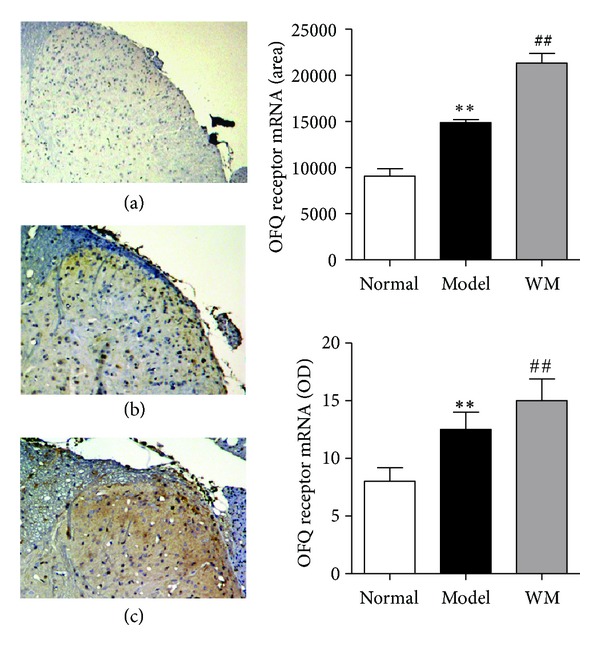
Effects of WM on the expression of OFQ receptor mRNA in L6-S1 spinal cord. (a) Spinal dorsal horn from normal group (×100). (b) Spinal dorsal horn from model group (×100). (c) Spinal dorsal horn from WM group (×100). Data are presented as the mean ± SEM (*n* = 6 per group). **P < 0.01 versus normal group; ^##^ *P* < 0.01 versus model group. WM: warming moxibustion.

The expression of OFQ and of its receptor in L6-S1 spinal dorsal horn was also tested by ISH, immunohistochemistry and ELISA. As shown in Figure 7, ISH examination revealed that the expression of OFQ mRNA in model group was higher than that in normal group (*P* < 0.01), and WM treatment further produced a marked elevation of OFQ mRNA (*P* < 0.01). As shown in Figure 8, the level of OFQ protein was higher in model group than that in normal group (*P* < 0.05), and significantly increased after WM treatment (*P* < 0.05). ISH showed that the expression of spinal OFQ receptor mRNA increased during CVH and was further remarkably enhanced by WM treatment (*P* < 0.01) (Figure 9). These data indicated that WM treatment induced the expression of OFQ and enhanced the transcription of OFQ receptor mRNA in CVH rats.

## 4. Discussion

Moxibustion has been adopted as an analgesic method for thousands of years in China, and it originated in the application of fire and then continuously developed in practice [31]. The primitive men, living in damp-cold caves, were suffering from pain in joints, and the symptoms could be relieved by basting in sunshine or fire; pressed by heated sand or soil, abdomen could be relieved from pain; applied fire to a certain part of the skin, toothache and stomachache could be attenuated. All of these archaeological investigations were the embryonic form of moxibustion therapy and also the beginning of moxibustion analgesia [32]. It has been shown that moxibustion could effectively treat human primary dysmenorrheal [33], knee osteoarthritis [34], rheumatoid arthritis [35], and cancer pain [36]. However, the mechanisms of moxibustion analgesia are not elucidated.

In the present study, the behavioral results showed that 7 times of WM at ST25 and ST37 significantly attenuated CRD-induced CVH in IBS-like rats, which is consistent with our previous studies [10–14]. It should be mentioned that WM treatment in the present and previous animal studies only partially inhibited CVH. This may be due to the fact that WM treatment in animal studies only involves stimulation of two acupoints, which also supports the clinical usage of multiple acupoints.

Here, the behavioral results demonstrated that intrathecal administration of dynorphinA (1-17) in analgesic dose relieved CVH, and *κ* receptor antagonist reversed the antihyperalgesic effect, indicating that dynorphinA (1-17) acts on *κ* receptors to impede nociceptive signals at the spinal level in this rat model. The expression of dynorphin in the superficial dorsal horn was found to be increased in both mRNA and protein levels in model rats, and the data also indicated that KOR mRNA transcription was induced in CHV. This suggests that spinal dynorphin-*κ* system could be an initiated exposure to nociceptive stimuli in CVH rats. It was reported that the increases of endogenous analgesic substances during pain are important to maintain homeostasis [37]. Thus, although dynorphin-*κ* system is known to be an antihyperalgesic agent, it may be upregulated following exposure to the nociceptive stimulus. This result is in accordance with studies that demonstrated that the expression of antihyperalgesic agents, such as neurotrophin-3, cannabinoid receptor-2, and interleukin-10, is significantly increased in rats with pain [37].

However, our pilot study employed ELISA to determine spinal dynorphin and found a decreased dynorphin conflict to the present result in CVH rats [13]. Then, we again adopted ELISA to determine the concentration of the dynorphin. The data showed that dynorphin concentration in L6-S1 spinal cord was largely increased in model group than that in normal group, which was in favor of the morphological results. The decreased dynorphin level in the pilot study might be due to a comparative shortage of dynorphin proteins produced by the imbalance between release and synthesis.

We also found that WM's analgesic effects in CVH rats were enhanced by i.t. dynorphinA (1-17) and partly attenuated following *κ* antagonist intervention, indicating the involvement of spinal that dynorphin-*κ* system in moxibustion analgesia. Also, the data showed dynorphin mRNA/protein and KOR mRNA were largely increased following 7 times of WM treatment, indicating that WM relieved visceral pain by activating spinal dynorphin-*κ* system.

Notably, regarding the behavioral results in Figure 2(b), the AWR score was increased under the i.t. *κ* antagonist intervention but without statistical significance at 5.32 KPa stimuli, and the AWR score was reduced under dynorphinA (1-17) intervention but without statistical significance at 10.64 KPa stimuli. In our opinion, this change may be due to the dose of dynorphinA (1-17) and *κ* antagonist.

In agreement with our findings, numerous studies demonstrate that dynorphin-*κ* system acts in an antinociceptive manner in the spinal cord [38–40]. However, conflicting reports showed that *κ* receptor cannot modulate visceral nociceptive transmission at the spinal level [41, 42]. Causes of these conflicting reports are still unclear. Different rat models of visceral pain and the use of kappa receptor agonist may be involved, so further investigation would be necessary.

The outcome of research on dynorphin-*κ* system inspired our study on OFQ-OFQ receptor system, and we presumed that the effect of moxibustion analgesia might be due to the induction of OFQ-OFQ receptor system in the spinal cord. The present study provides the convincing evidence for our presumption, and we found that WM's analgesic effects in CVH rats were enhanced by intrathecal administration of OFQ and partly attenuated following OFQ receptor antagonist, indicating the involvement of spinal OFQ-OFQ receptor system in moxibustion analgesia. Also, the data showed that OFQ and OFQ receptor mRNA were largely increased following 7 times of WM treatment, indicating that WM may relieve visceral pain by activating spinal OFQ-OFQ receptor system.

Taken together and based on the mechanism of acupuncture analgesia, we infer further that moxibustion at ST 37 and ST 25 could stimulate the special receptors in acupoints and produce heat signals projected to CNS; in the process of transmission, the heat signals may partly block the input of pain sensation in the spinal dorsal horn by activating the segmental inhibition (including both presynaptic and postsynaptic inhibition) or initiating the descending inhibition system, or both. The integrated action of segmental inhibition and descending inhibition may activate the spinal dynorphin and OFQ system, modulate the release of excitatory neurotransmitter in primary afferent fibers, and suppress the response of spinal neurons to the noxious stimuli. The pathway of the thermal stimulation signal is similar to that of algesic signal [43], which provides a morphological basis for the interaction of the two signals. The neural mechanisms of moxibustion analgesia should be complicated and it is possible that moxibustion may modulate the activities of various peptides or neurotransmitters to inhibit pain. However, more concrete evidence is required from future investigations. 

## 5. Conclusions

Our results demonstrate that WM treatment may attenuate CRD-induced chronic visceral hyperalgesia in IBS-like rats and that this effect may be due, at least in part, to WM induction of spinal dynorphin and orphanin-FQ system. 

## Figures and Tables

**Table 1:** AWR scoring criteria.

Score 0	No behavioral response to CRD
Score 1	Immobile during the CRD and occasionally clicked the head at the onset of the stimulus
Score 2	A mild contraction of the abdominal muscles, but no lifting the abdomen off the platform
Score 3	A strong contraction of the abdominal muscles and lifting the abdomen off the platform, no lifting the pelvic structure off the platform
Score 4	Arching body and lifting the pelvic structure and scrotum

AWR: abdominal withdrawal reflex; CRD: colorectal distention.
